# ADVANCES IN GLOBAL AGING RESEARCH FROM CROSS NATIONAL COMPARISONS

**DOI:** 10.1093/geroni/igad104.0944

**Published:** 2023-12-21

**Authors:** Emma Nichols, Jennifer Ailshire

## Abstract

Harmonized surveys on aging populations around the world provide an unprecedented opportunity to advance our understanding of the factors that promote healthy aging. The papers in this symposium use existing and newly available harmonized data from the NIA-funded Gateway to Global Aging to examine how patterns of physical and cognitive aging vary across countries and to identify potential modifiable risk factors for slowing aging-related decline. Jayakody et al. use data on 21 countries to identify risk factors for Motoric Cognitive Risk (MCR), a preclinical predictor of dementia, that are both country-specific and universal. Knapp et al. also investigate potential modifiable risk factors for poor cognitive health in 31 countries and identify the factors that are universally associated with higher risk across countries. These two studies provide novel insights into how we can harness studies across multiple countries to better understand which factors of aging are universal and which are context dependent. The next two studies examine patterns and risks through comparison of neighboring countries. Focusing on Mexico and Brazil, Gomes Goncalves et al., determine the extent to which rurality and education confer risk of cognitive impairment in the two largest countries in Latin America. Finally, Fermín-Martínez et al. develop a new measure to capture age-related functional decline that focuses on anthropometric information available in aging surveys, and they validate their measure in older adults in the United States and Mexico. The papers in this symposium show how cross-national comparisons are being used to advance our understanding of aging.

